# Intelligent Vehicle Target Detection Algorithm Based on Multiscale Features

**DOI:** 10.3390/s25165084

**Published:** 2025-08-15

**Authors:** Aijuan Li, Xiangsen Ning, Máté Zöldy, Jiaqi Chen, Guangpeng Xu

**Affiliations:** 1 School of Automotive Engineering, Shandong Jiaotong University, Jinan 250357, China; 13933028705@163.com (X.N.); chenjiaqi@stu.sdjtu.edu.cn (J.C.); cyxu225@163.com (G.X.); 2 Department of Environmental Economics and Sustainability, Faculty of Economics and Social Sciences, Budapest University of Technology and Economics, Műegyetem rkp 3-5, H-1111 Budapest, Hungary; zoldy.mate@gtk.bme.hu

**Keywords:** YOLOv10, intelligent vehicle, target detection, multi-scale flexible convolution, shallow auxiliary fusion

## Abstract

To address the issues of false detections and missed detections in object detection for intelligent driving scenarios, this study focuses on optimizing the YOLOv10 algorithm to reduce model complexity while enhancing detection accuracy. The method involves three key improvements. First, it involves the design of multi-scale flexible convolution (MSFC), which can capture multi-scale information simultaneously, thereby reducing network stacking and computational load. Second, it reconstructs the neck network structure by incorporating Shallow Auxiliary Fusion (SAF) and Advanced Auxiliary Fusion (AAF), enabling better capture of multi-scale features of objects. Third, it improves the detection head through the combination of multi-scale convolution and channel adaptive attention mechanism, enhancing the diversity and accuracy of feature extraction. Results show that the improved YOLOv10 model has a size of 13.4 MB, meaning a reduction of 11.8%, and that the detection accuracy mAP@0.5 reaches 93.0%, outperforming mainstream models in comprehensive performance. This work provides a detection framework for intelligent driving scenarios, balancing accuracy and model size.

## 1. Introduction

Object detection is the cornerstone of intelligent driving systems [1], enabling vehicles to perceive obstacles, pedestrians, and traffic signs around them in real time and respond accordingly. However, the complex road environment, characterized by different object scales and severe occlusion, poses significant challenges to the existing models [2]. This often leads to incorrect detection, missed detection, and low deployment efficiency on resource-limited in-vehicle hardware.

Traditional object detection methods rely on manual feature extraction [3], which cannot capture deep semantic information and limits performance in complex scenarios. The emergence of deep learning has completely transformed this field. Single-stage detectors like YOLO [4] and SSD [5] have become leaders due to the balance they strike between speed and accuracy. Since its launch in 2016, YOLO has undergone rapid iteration, continuously optimizing accuracy and latency in practical applications. According to recent research results, many applications for YOLOv10 have been explored in object detection. Du Hong et al. [6] proposed a YOLOV10-Vehicle object detection algorithm. A new attention mechanism module, WT-PSA, was designed to enhance the model’s attention to vehicle targets in complex weather conditions. Chen Haixiu et al. [7] proposed an improved pedestrian small-target detection algorithm based on YOLOv10n. This algorithm integrates adaptive point movement and convolutional gating mechanisms into the C2f module, significantly enhancing its ability to capture local features and improving feature screening. Junhui Mei et al. [8] proposed a lightweight object detection algorithm, BGF-YOLOv10, which combines variants of BoTNet, C2f, and C3 in the backbone network to enhance detection performance regarding small objects. However, even the latest version still struggles with small targets and partially occluded vehicles in heavy traffic and has difficulties with multi-scale object representation and occlusion processing.

Against this backdrop, the limitations of current methods in intelligent driving scenarios are evident:

(1) Environmental complexity degrades feature quality, increasing detection errors.
(2) Extreme scale variations—from distant cyclists to nearby trucks—strain fixed-receptive-field convolutions, reducing localization accuracy.
(3) Severe occlusions obscure critical features, leading to missed or misclassified targets.

To address these issues, this study proposes targeted improvements to YOLOv10s, aiming to enhance detection precision while reducing model complexity. The core contributions are threefold:

(1) A multi-scale flexible convolution (MSFC) to dynamically adapt to varying feature scales, reducing computational overhead.
(2) A reconstructed neck network integrating Shallow Auxiliary Fusion (SAF) and Advanced Auxiliary Fusion (AAF) to optimize multi-scale feature interaction.
(3) A SEAM, combining multi-scale convolutions and channel attention, to boost feature extraction robustness.

These innovations collectively mitigate the limitations of existing models in complex traffic scenarios, paving the way for more-reliable intelligent driving perception systems.

The structure of this article is as follows: Section 1 introduces the background, limitations of existing methods, and core contributions of the study. Section 2 reviews related work on YOLO series evolution and YOLOv10 research. Section 3 details the improved YOLOv10 target detection algorithm and its key components. Section 4 details the training and validation of the model, as well as the comparison and ablation experiment to verify the effectiveness of the algorithm in this paper. Section 5 discusses the study’s results, limitations, and future directions. And Section 6 draws conclusions to summarize the whole paper.

## 2. Related Work

### 2.1. Target Detection Method

The application of object detection technology in the field of intelligent driving needs to simultaneously meet the three core requirements of real-time performance, multi-scale adaptability, and anti-interference ability [9]. At present, the mainstream methods can be classified into two-stage detectors, single-stage detectors, and anchor-free detectors, etc. Each type of method has specific advantages and limitations in intelligent driving scenarios. Two-stage detectors (such as Faster R-CNN [10] and Mask R-CNN [11]) have relatively high accuracy, but they have slow reasoning speed and large computational load, making it difficult to adapt to real-time scenarios. Single-stage detectors (such as SSD [5] and Retina Net [12]) are more efficient, but they have problems such as weak detection of small targets and poor adaptability to occluded scenes. Anchor-free methods (such as Center Net [13] and FCOS [14]) simplify the network but are sensitive to scale changes and prone to false detections in dense scenarios. Although lightweight models (such as Mobile Net-SSD [15]) are compatible with the hardware, their accuracy loss is significant in complex road conditions. The YOLO series, as a representative of single-stage detectors, is widely used in intelligent driving.

### 2.2. Evolution of YOLO Series in Intelligent Driving

YOLO has become a dominant choice for real-time object detection in intelligent driving due to its efficiency. YOLOv1 [16] pioneered the grid-based single-stage approach but struggled with small target detection. Subsequent iterations addressed this: YOLOv3 [17] introduced multi-scale prediction via feature pyramids, improving small target recall; YOLOv5 [18] optimized backbone and neck structures, reducing model size while enhancing accuracy; YOLOv7 [19] integrated trainable bag-of-freebies techniques, boosting performance on traffic datasets like BDD100k. YOLOv10 [20], the latest iteration, achieves state-of-the-art speed–accuracy tradeoffs, provides higher detection accuracy while maintaining lower latency compared to existing real-time end-to-end detectors, and represents the latest advancement in real-time target detection.

### 2.3. Research on the Object Detection Algorithm of YOLOv10

YOLOv10 is a next-generation real-time end-to-end target detection model proposed in May 2024 by a team from Tsinghua University. Compared with previous generations of YOLO detection algorithms, YOLOv10 achieves significant computational accuracy tradeoffs across model sizes [21], but its fixed convolution kernels and conventional neck design still restrict adaptability in complex road environments. This paper makes targeted improvements. It designs multi-scale flexible convolution (MSFC), reconstructs the neck network structure to include Shallow Auxiliary Fusion (SAF) and Advanced Auxiliary Fusion (AAF), and introduces the SEAM module to enhance the focus of the detection head. While maintaining a lightweight model size (13.4 MB), it enhances the detection robustness in complex traffic scenarios.

## 3. Methodology

### 3.1. Overall Architecture of Improved YOLOv10

**Figure 1:**
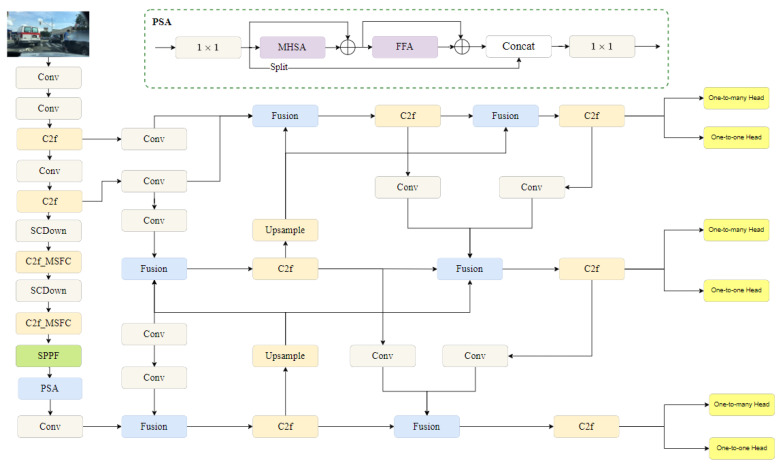
Improved YOLOv10s network structure diagram.

Due to the limited performance of current intelligent vehicles’ on-board computers, higher requirements are placed on the deployed model, i.e., minimizing model size while ensuring higher accuracy and real-time performance. Therefore, in this paper, YOLOv10s is used as the base target detection algorithm to meet the initial requirements of hardware conditions and detection performance of intelligent vehicles. However, the base model still has some room for improvement in terms of model complexity, accuracy of small and occluded targets. Aiming at the above problems. In this paper, YOLOv10s is improved by designing multi-scale flexible convolution (MSFC), reconstructing the neck network structure to include Shallow Auxiliary Fusion (SAF) and Advanced Auxiliary Fusion (AAF), and improving the detection head (SEAM) that combines multi-scale convolution and a channel adaptive attention mechanism. In Figure 1, the network structure diagram of the improved YOLOv10s is presented to better meet the requirements of intelligent vehicle target detection and provide more-accurate detection information for subsequent target tracking.

### 3.2. Design of Multi-Scale Flexible Convolution

Traditional convolutional layers usually use a fixed convolutional kernel size (e.g., 3 × 3 or 5 × 5). The limitations of this fixed sense field mean the convolution operation is not flexible enough to face different scales of features. When the size of the input image or the feature size difference is large, the traditional convolution needs to be stacked several times in order to capture different sizes of the features, which increases the amount of computation. In addition, in the face of complex traffic conditions, the feature size varies greatly, and the same convolutional kernel cannot adapt to features of different sizes, resulting in unsatisfactory detection results.

In response to this issue, this paper proposes MSFC, whose mathematical expression is shown in Equation (1).

(1) $$y=\sigma (W_{1}\cdot {Conv}_{3\times 3}(x_{1})+W_{2}\cdot {Conv}_{5\times 5}(x_{2})+W_{0}\cdot {Conv}_{1\times 1}(x))$$

where $x=x_{1}⊕x_{2}$; W_1_, W_2_, and W_3_ are learnable fusion weights that satisfy W_1_ + W_2_ + W_3_ = 1.

**Figure 2:**
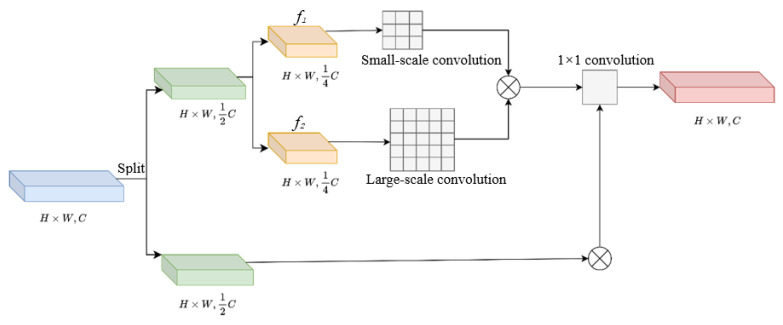
Schematic diagram of multi-scale flexible convolution structure.

The structural schematic diagram of multi-scale flexible convolution is shown in Figure 2. A schematic diagram of the structure of multi-scale flexible convolution, which uses multiple different scales to process input features, can simultaneously capture information from different scales and fuse it with 1 × 1 convolution to fully integrate multi-scale information, reduce network stacking, and lower the computational load. The input is a feature map of size H × W with channel number C. After the segmentation operation, two feature maps with halved channel numbers are obtained and named *f*_1_ and *f*_2_, respectively. Two convolutions of different scales were performed on *f*_1_ and then concatenated together. Through a 1 × 1 convolutional layer, the features of different scales were integrated to obtain the same result as the *f*_1_ features, resulting in an output of size H × W with channel number C.

As a key feature fusion module in YOLOv10, the original C2f [22] excels in balancing feature extraction efficiency and representational capability through its dual-path structure. Although MSFC can extract multi-scale features more effectively, it lacks layer-by-layer feature fusion and hierarchical information transfer. To compensate for this, this paper chooses to combine it with the improved C2f module to leverage the advantages of both and optimize the information flow and transfer through multiple feature fusion, so as to enhance the model’s representation capability and performance.

**Figure 3:**
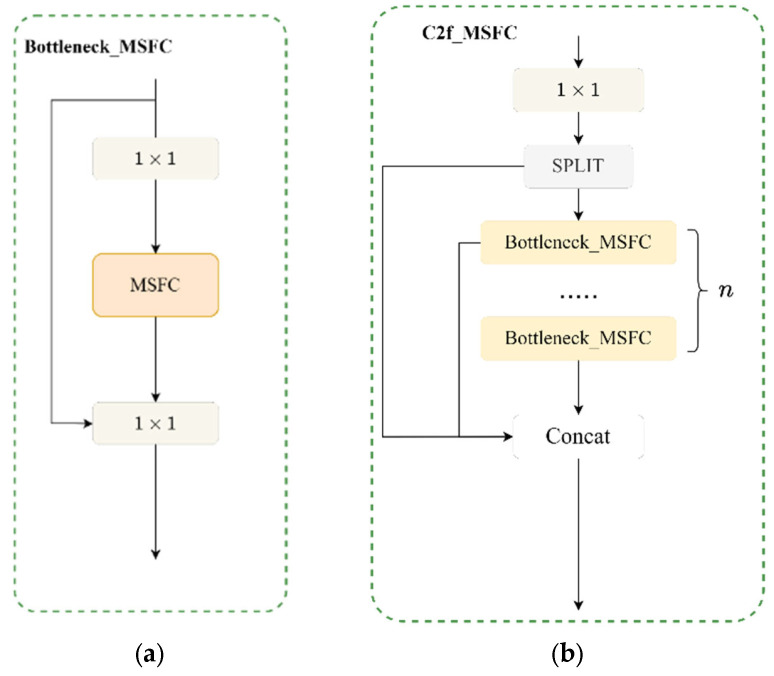
MSFC structure embedded in other modules. (**a**) Bottleneck_MSFC, (**b**) C2f_MSFC.

In this paper, the lightweight Bottleneck_MSFC module is first designed to replace the Bottleneck module in the YOLOv10s network, and its structure is shown in Figure 3a. On this basis, this paper improves the C2f module by using MSFC-Bottleneck instead of the original Bottleneck, and designs the C2f-Bottleneck module, whose structure is shown in Figure 3b.

### 3.3. Design of Neck Network

The main goal of Feature Pyramid Network (FPN) is to solve the problem of information loss and resolution mismatch in target detection and segmentation at different scales. At first, it was proposed by Lin et al. [23] in 2016 and applied in the Faster R-CNN detector; then, based on this, Liu et al. [24] proposed PAN structure to solve the problem of information loss and incompleteness that may exist in the feature fusion process of FPN. Yang et al. [25] proposed a novel structure of Multi-Branch Auxiliary FPN (MAFPN). MAFPN achieves a richer feature fusion by introducing the two key modules of Superficial Assisted Fusion (SAF) and Advanced Assisted Fusion (AAF). By introducing these two key modules, MAFPN realizes richer feature interactions and fusion and effectively improves the detection performance of the model for targets at different scales.

#### 3.3.1. Superficial Assisted Fusion (SAF)

**Figure 4:**
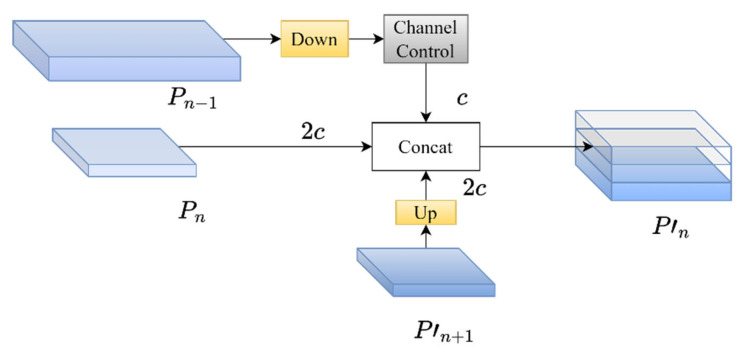
Schematic diagram of SAF module structure.

The SAF module is a key component in MAFPN for preserving shallow spatial information. The “shallow features” referred to in this article mean the feature maps in neural networks that are close to the input layer. It features higher spatial resolution and richer detailed information. However, the semantic information is relatively weak and is mainly used for target positioning. “Deep features” refer to the feature maps close to the output layer, which have relatively low spatial resolution but rich semantic information and are mainly used for target classification. In the task of object detection, the shallow features provided by the shallow network contain rich edge information and detailed features, which are crucial for the precise localization of small targets. Traditional feature fusion methods often fail to preserve such shallow information during the fusion process. To overcome this problem, MAFPN designed the SAF module, incorporating shallow information as an auxiliary branch into the deep network. As shown in the schematic diagram of the SAF module structure in Figure 4, the SAF module is spliced with feature maps of different resolutions (e.g., P_n−1_, P_n_, P_n+1_). In the feature pyramid structure, the current layer P_n_ is taken as the reference. P_n−1_ represents the upper layer (belonging to shallow features), and P_n+1_ represents the lower layer (belonging to deep features). P_n−1_ and P_n+1_ are subjected to downsampling and upsampling operations, respectively, to ensure the spatial alignment of the feature map. In addition, this paper uses 1 × 1 convolution to control the number of channels of shallow information, making its proportion in the concatenation operation smaller and thus not affecting subsequent learning. By using the SAF module, MAFPN can retain more shallow spatial information in deep networks, significantly improving the detection accuracy of small targets and enhancing the spatial representation of the network.

#### 3.3.2. Advanced Assisted Fusion (AAF)

**Figure 5:**
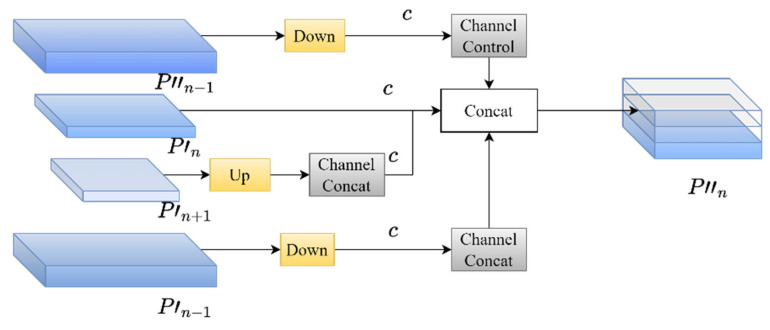
Schematic diagram of AAF module structure.

The AAF module is a key component in MAFPN used to further enhance the feature–level information interaction. In the target detection task, accurate classification relies on the coarse-grained information provided by the deep network, while accurate localization requires the detailed information provided by the shallow network. In order to realize the full fusion of shallow and deep information, MAFPN designs the AAF module to improve the model’s detection performance for medium-sized targets through the integration of multi-scale information. For specific implementation, the schematic structure of the AAF module is shown in Figure 5. AAF is achieved through multi-source feature aggregation, and the specific formula is shown in Equation (2).

(2) $$P_{i}^{AAF}={Conv}_{1\times 1}(Concat(P_{i},↓P_{i-1},↑P_{i+1},P_{i}^{sibling}))$$

where ↓ and ↑ represent downsampling and upsampling, respectively.

AAF performs multi-scale information integration in the deep layer of MAFPN, involving the aggregation of information from the shallow high-resolution layer ($P_{n+1}^{\prime }$) the shallow low-resolution layer ($P_{n-1}^{\prime }$), the sibling shallow layer (${P^{\prime }}_{n}$), and the previous layer (${P^{\prime \prime }}_{n-1}$).

In this way, the final output layer is able to fuse information from four different layers at the same time, which significantly improves the model’s ability to detect medium-sized targets. The AAF also employs a 1 × 1 convolution to regulate the influence of each layer on the output results, ensuring that the model is able to capture rich information from features at different scales. With the AAF module, the MAFPN is able to better capture the multi-scale features of the target, which improves the detection accuracy of medium-sized targets and enhances the feature representation capability of the model.

### 3.4. Detection Head Improvements

Conventional convolutional operations and feature fusion approaches may not be able to handle multi-scale features efficiently, thus limiting the model’s ability to perceive objects of different sizes. Existing detection heads usually do not have the flexibility to weight different channels in the feature map, which affects the performance of the model in complex scenes. To compensate for these shortcomings, this paper introduces an improved scheme based on SEAM [26] (separated and enhancement attention module), which aims to enhance the feature extraction capability and robustness of the model by introducing multi-scale convolution and channel attention mechanisms.

**Figure 6:**
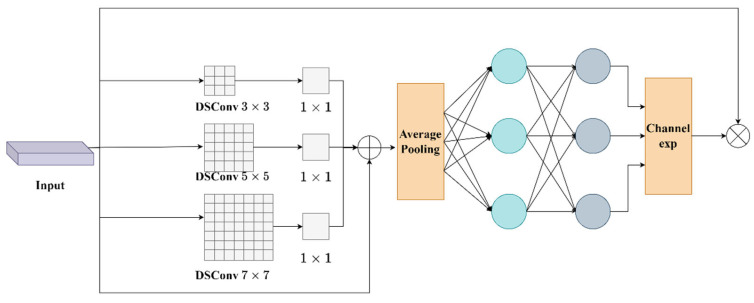
Schematic diagram of SEAM module structure.

As shown in the schematic structure of SEAM in Figure 6, the core idea behind the SEAM module is to combine multi-scale convolution and a channel adaptive attention mechanism to enhance the diversity and accuracy of feature extraction. SEAM employs multi-scale depth-separable convolution, as shown in Equation (3).

(3) $${F=\mathrm{Concat}\;(\mathrm{DSConv}}_{3\times 3}({x),\;\mathrm{DSConv}}_{5\times 5}({x),\;\mathrm{DSConv}}_{7\times 7}(x))$$

In this module, local features at different scales are extracted by first applying convolution operations to the input feature map using convolution kernels of different sizes. In this way, the model is able to capture feature information about the object from multiple scales of view, especially when dealing with targets of different sizes or with complex backgrounds, which can significantly improve the detection accuracy. Subsequently, SEAM decomposes the convolution operation into depth-wise convolution and pointwise convolution using depth-wise separable volume accumulation. While maintaining the efficiency of feature extraction, the number of parameters is reduced, thereby effectively lowering the computational overhead while ensuring performance.

In addition, SEAM introduces a channel attention mechanism that utilizes an adaptive approach to weight the importance of different channels. Specifically, the output of each channel is weighted through global average pooling and fully connected layers to adjust the contribution of each channel in the feature graph. This channel attention mechanism enables the network to pay more attention to features that are helpful for target recognition during the learning process, while suppressing the influence of noise and irrelevant features, thus improving the accuracy and robustness of target detection.

## 4. Experimentation and Analysis

### 4.1. Dataset Construction

**Figure 7:**
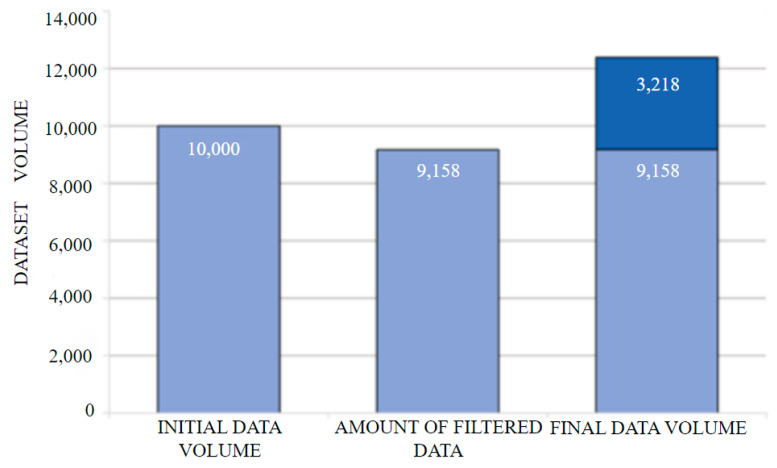
Comparison of dataset quantities before and after processing.

This study adopted a systematic data construction strategy, with the aim of creating a specialized vehicle inspection dataset. The original training data was derived from the fusion of the BDD100k [27] (Berkeley DeepDrive 100k) dataset with rich traffic scene semantics and the high-resolution street view dataset Cityscapes [28]. In view of the original annotation characteristics of the BDD100k dataset, we first implemented semantic annotation space reconstruction. We then performed annotation merging operations on the five types of transportation vehicles in the original annotations, namely “car”, “bus”, “truck”, and “motorcycle”, and uniformly marked them as included in the “car” category. Through Python script automated batch processing, multi-scale vehicles in a single image can obtain unified semantic identifiers. To address the limitations of single-dataset scenarios, left-view RGB images from the Cityscapes dataset were introduced for cross-dataset fusion. The format conversion of bounding box annotations was then completed using the LabelImg tool. A total of 3218 urban scene images containing multi-angle vehicle entities were selected. After supplementation, the total number of the database expanded to 12,367, representing an increase of 35.1% compared to the original BDD100k vehicle-related data volume. The comparison of the number of datasets is shown in Figure 7, which compares the number of datasets before and after processing.

To guarantee the generalization performance of the model, the data was divided by random sampling: 20% of the data was reserved for the test set, and the remaining 80% was split into the training set and validation set by 8:2.

### 4.2. Model Training and Experimental Validation

In order to test the performance of the proposed algorithm, the above expanded dataset was used as the experimental dataset for training and validation, and the hardware configuration parameters, software environment parameters, and experimental parameters of the experiment are shown in Table 1 (Experimental Hardware Configuration Parameters), Table 2 (Experimental Software Environment Parameters), and Table 3 (Experimental Parameter Settings), respectively.

In order to verify the performance of the improvement algorithms proposed in this paper and evaluate the effect of different modules and the interaction between different modules, we designed eight groups of ablation experiments to verify the effectiveness of each improvement strategy using YOLOv10s as a baseline model, named A–H, and the meanings represented by the names of each serial number are shown in Table 4 (Meaning of each serial number name in the ablation experiment). “√” indicates that this module exists in this group of experiments. Among them, A represents the original YOLOv10s, and B to H are the combined schemes incorporating MSFC, MAFPN, and SEAM modules.

From the results of the ablation experiments in Table 5, it can be seen that the experimental group B, which adds the MSFC module to the baseline model, shows a large reduction in both the model computation and the number of parameters, which proves the effectiveness of the model in lightweighting; the experimental group C, which adds the MAFPN structure, shows the same reduction in the number of parameters, the size of the model is decreased by 2 MB, and the accuracies, mAP50 and mAP50-95, are both improved, indicating that the network structure can realize the functions of accuracy improvement and lightweighting at the same time. Experimental group D adds SEAM to the detection head, which causes a greater improvement in accuracy but, at the same time, brings about a significant increase in model size. Group E simultaneously introduces multi-scale flexible convolution (MSFC) and an improved neck network (MAFPN). The experimental results show that the model parameters (6.40 M) and computational load (20.7GFlOPs) are significantly lower than those of the baseline model A. The model size was compressed to 13.4 MB, and the mAP50 reached 91.7%. The reduced parameters of MSFC provide computational redundancy space for the feature fusion of MAFPN. The hierarchical fusion mechanism of MAFPN makes up for the deficiency of MSFC in deep feature transfer. Eventually, alongside a 19% reduction in parameters, mAP50 improved by 0.6% compared to the baseline. Group F added MSFC and an improved detection head (SEAM) on the basis of the baseline model, increasing mAP50 to 93.2%. However, the model parameters of 13.00 M and the computational load of 21.1GFlOPs have increased. The lightweight feature of MSFC partially offsets the increase in parameters brought by SEAM. The model size is 15.8 MB, only 1.1 MB larger than the baseline, while maintaining a high real-time performance of 139FPS. However, due to the depth-wise separable convolution and attention mechanism of the SEAM module, additional computational branches are still introduced. This led to the total number of parameters being higher than that of group B, which only contained MSFC, verifying the suggestion that improvements in accuracy come at the cost of a moderate increase in parameters. Group G, combining MAFPN and SEAM, achieved a mAP50 of 93.5%, making it the group with the highest accuracy among all combinations except group H. However, the model parameters of 12.13 M and the computational load of 21.8GFlOPs are still relatively high. The enhancement of medium target features by the AAF module complements the multi-scale convolution of SEAM. MAFPN enhances the spatial correlation of features, while SEAM strengthens the discriminability of channel dimensions. The two work together to increase mAP50-95 by 1.3% compared to the baseline. However, due to the non-introduction of the lightweight design of MSFC, the model parameters are still higher than those of group E. This indicates that although the combination of feature fusion and attention mechanisms can maximize accuracy, the computational cost needs to be balanced through lightweight modules such as MSFC.

After integrating MSFC and MAFPN, group H effectively mitigates the larger parameter count and model size introduced by SEAM and achieves a balance between accuracy and lightness, with mAP50 and mAP50-95 improved by 1.9 and 1.7 percentage points, and arithmetic and model sizes reduced by 3.5 and 2.3 percentage points, respectively.

In order to show the advantages of this paper’s algorithm in comparison with other mainstream algorithms of the same volume, seven groups of comparison experiments were conducted for this paper, and the results of the experiments are shown in Table 6. In the comparison of mAP50 and mAP50-95 evaluation metrics, the algorithm proposed in this paper shows an advantage in the seven sets of comparison experiments; its results are 1.4 and 0.5 percentage points higher than that of yolov10m in the higher level, respectively. In terms of the number of parameters, that of this paper’s algorithm is higher than the baseline model due to the improvement of the detector head on the basis of yolov10s, which has increased the parameter number by a certain amount, but it is still lower than that of yolov10m. In terms of the number of operations, that of this paper’s algorithm is higher than the baseline model because of the improvement in detection head based on yolov10s and yolov10m. In terms of computing power, that of this paper’s algorithm is 3.5GFlops lower than that of the baseline model, and the model size is the smallest except for yolov7-tiny. Although the FPS is reduced after the improvement, the real-time performance can still be maintained at a high level. The comparison of experimental results shows that the improved algorithm proposed in this paper, compared with other mainstream models of the same level, shows certain advantages in most of the evaluation indicators.

**Figure 8:**
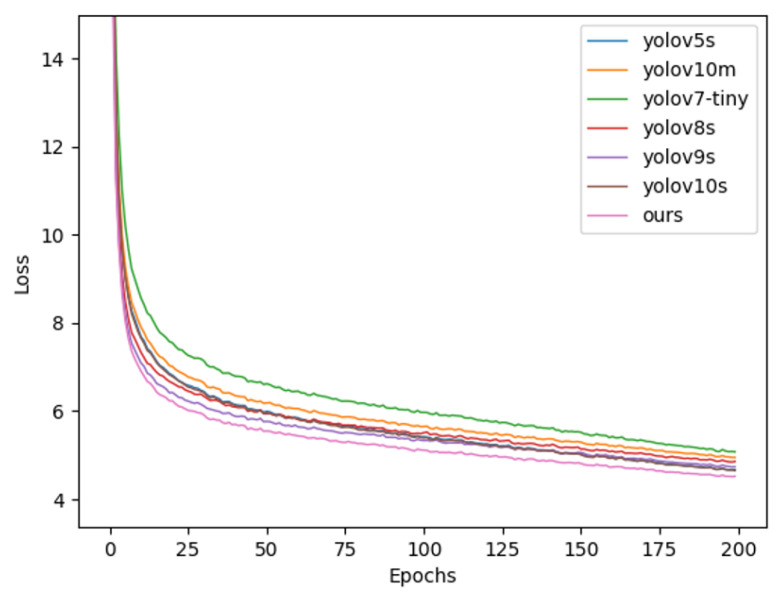
Comparison of the loss results of the comparison experiment.

**Figure 9:**
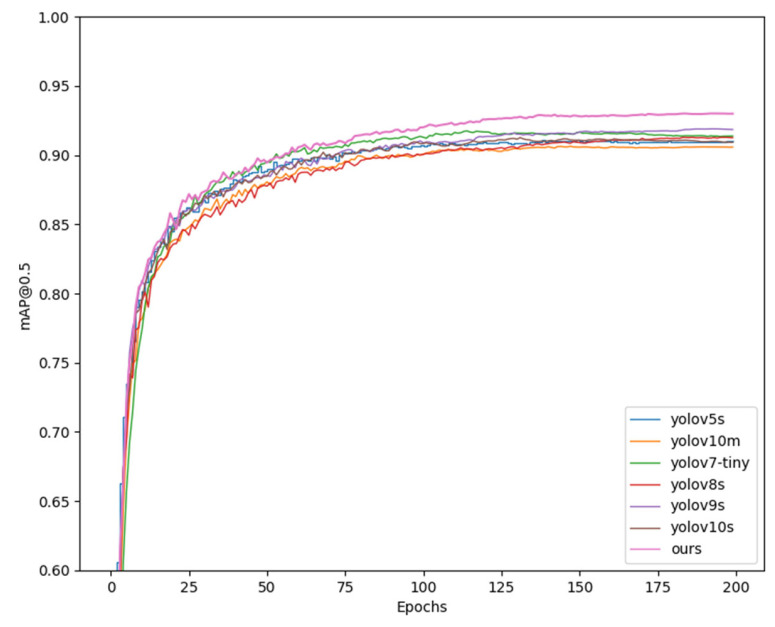
Comparison of the mAP@0.5 results of the comparison experiment.

**Figure 10:**
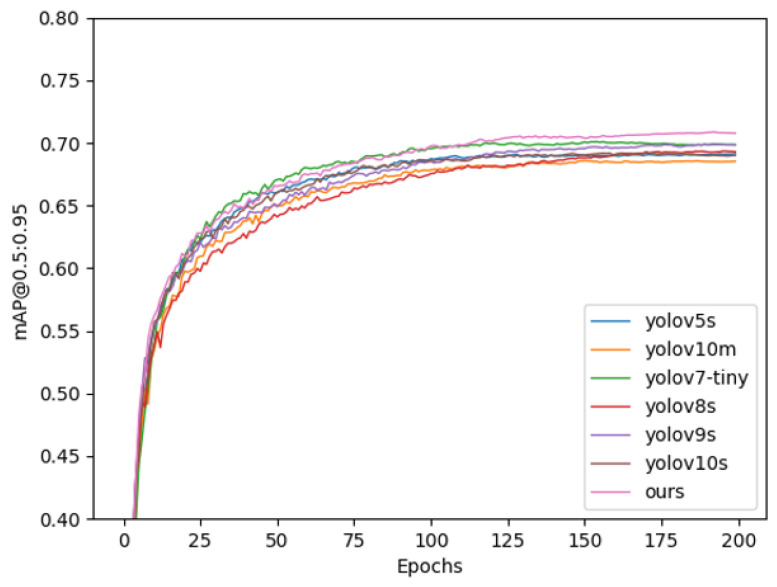
Comparison of the mAP@0.5:0.95 results of the comparison experiment.

In order to visually display the results of the comparison experiment, seven sets of comparison experiments and mAP comparison diagrams were drawn. Figure 8 (Comparative experimental loss results comparison chart) shows the loss results of seven groups of comparison experiments. When the experiments are all converged, the algorithm in this paper can obtain the lowest loss results; Figure 9 (Comparative experimental mAP@0.5 results comparison chart) shows the comparison results of the evaluation index mAP@0.5; Figure 10 (Comparative experimental mAP@0.5:0.95 results comparison chart) shows the results of mAP@0.5:0.95, and the comparison charts show that the algorithm (pink curve) in this paper has an advantage over mainstream algorithms.

## 5. Discussion

In this study, the improved YOLOv10 model addresses the key challenges in intelligent driving target detection by introducing MSFC, MAFPN, and SEAM. Experimental results prove that these improvements effectively balance detection accuracy and model size, and the algorithm outperforms mainstream baselines such as YOLOv10s, YOLOv9s, and YOLOv8s (93.0%) regarding mAP@0.5, while reducing the model size to 13.4 MB. These results are consistent with the core assumption that optimizing multi-scale feature extraction, neck network information flow, and the adaptability of the detection head can enhance performance in complex traffic scenarios.

However, this study also has limitations. Firstly, the datasets from BDD100k and Cityscape mainly focus on urban road scenarios, which may limit the application of the results under extreme conditions (such as off-road environments, heavy fog or snow, and other bad weather), where the degradation of sensor data is more obvious. Secondly, although the model size was compressed, the FPS dropped to 128, which may not fully meet the ultra-low latency requirements of real-time autonomous driving systems, especially for high-speed decision-making tasks. Thirdly, although SEAM improves accuracy, it introduces additional computational overhead, and its efficiency in dynamic traffic with rapidly changing target densities still needs to be studied.

Future research can focus on two aspects: First, expanding the dataset to include different scenarios (such as rural roads, bad weather) will enhance the robustness of the model. Future work will also explore lightweight focus mechanisms or model quantization techniques to further enhance inference speed without sacrificing accuracy and to ensure compatibility with in-vehicle edge computing devices.

## 6. Conclusions

To address issues of false detections, missed detections, and low deployment efficiency in intelligent vehicle target detection within complex traffic scenarios, this study optimized YOLOv10s to balance detection accuracy and model lightweighting. Key improvements include designing multi-scale flexible convolution (MSFC) to synchronously extract multi-scale features; reducing computational costs and enhancing representation of targets across scales; reconstructing the neck network with Shallow Auxiliary Fusion (SAF) and Advanced Auxiliary Fusion (AAF) for richer feature fusion; improving small and medium target detection; and proposing SEAM, which integrates multi-scale convolution and channel adaptive attention, to enhance feature extraction robustness. Experimental results on the fused BDD100k and Cityscapes dataset show that the improved model achieves a mAP@0.5 of 93.0%, outperforming mainstream models like YOLOv10s and YOLOv9s, while compressing the model size to 13.4 MB (an 11.8% reduction from the baseline). This work provides a high-performance solution for intelligent driving, laying the groundwork for in-vehicle embedded deployment. Future research will focus on expanding datasets to include diverse scenarios to enhance model robustness, as well as exploring lightweight attention mechanisms or model quantization to further improve inference speed, ensuring compatibility with in-vehicle edge computing devices.

## Figures and Tables

**Table 1:** Experimental hardware configuration parameters.

Parameters	Configuration
CPU	Intel i5-13600KF CPU@ 3.5 GHz
RAM	32G
GPU	NVIDIA GeForce RTX 4070Ti SUPER
Display Memory	16G

**Table 2:** Experimental software environment parameters.

Virtualized Environment	Anaconda 3.0
Language	Python 3.9
CUDA	12.3
Deep Learning Frame	PyTorch 1.12.0
OpenCV	4.9.0
OS	Windows 10

**Table 3:** Experimental parameter settings.

Parameters	Configuration
Epochs	200
Batch size	8
Learning rate	0.0001
Image size	640 × 640

**Table 4:** Meaning of each serial number name in the ablation experiment.

Label	YOLOv10s	MSFC	MAFPN	SEAM
A	√			
B	√	√		
C	√		√	
D	√			√
E	√	√	√	
F	√	√		√
G	√		√	√
H (ours)	√	√	√	√

**Table 5:** Results of ablation experiments.

Experiments	mAP50 (%)	mAP50-95 (%)	P (%)	R (%)	FlOPs (G)	Params (M)	FPS (f/s)	Model Size (MB)
A	91.1	69.2	91.2	83.1	24.4	8.04	270	15.7
B	91.4	69.1	90.8	82.9	21.0	7.84	227	16.9
C	91.9	69.9	92.2	82.7	21.7	6.98	244	14.5
D	93.8	71.2	93.4	85.6	22.3	13.26	151	27.3
E	91.7	69.9	91.4	82.8	20.7	6.40	196	13.4
F	93.2	70.5	90.1	82.2	21.1	13.00	139	15.8
G	93.5	70.5	90.4	83.1	21.8	12.13	135	15.5
H (ours)	93	70.9	92.4	82.8	20.9	11.56	128	13.4

**Table 6:** Comparison of experimental results.

Detectors	mAP50 (%)	mAP50-95 (%)	P (%)	R (%)	Params (M)	FlOPs (G)	Model Size (MB)	FPS (f/s)
yolov10s	91.1	69.2	91.2	83.1	8.04	24.4	15.7	270
yolov10m	91.6	70.5	91.9	83.2	15.31	63.4	31.9	250
yolov9s	91.6	70.3	91.3	83.1	7.17	26.7	15.2	222
yolov8s	91.3	69.2	90.3	84.1	11.1	22.5	21.4	238
yolov7-tiny	90.5	69.0	91.1	83.1	6.0	13.0	12.3	276
yolov5s	91.1	69.2	92.8	81.3	9.11	23.8	18.5	256
ours	93	70.9	92.4	82.8	11.56	20.9	13.4	128

## Data Availability

Data are contained within the article.
